# Prognosis and improved outcomes in major depression: a review

**DOI:** 10.1038/s41398-019-0460-3

**Published:** 2019-04-03

**Authors:** Christoph Kraus, Bashkim Kadriu, Rupert Lanzenberger, Carlos A. Zarate Jr., Siegfried Kasper

**Affiliations:** 1 Department of Psychiatry and Psychotherapy, Medical University of Vienna, Vienna, Austria; 2 Section on Neurobiology and Treatment of Mood Disorders, Intramural Research Program, National Institute of Mental Health, National Institutes of Health, Bethesda, MD USA

## Abstract

Treatment outcomes for major depressive disorder (MDD) need to be improved. Presently, no clinically relevant tools have been established for stratifying subgroups or predicting outcomes. This literature review sought to investigate factors closely linked to outcome and summarize existing and novel strategies for improvement. The results show that early recognition and treatment are crucial, as duration of untreated depression correlates with worse outcomes. Early improvement is associated with response and remission, while comorbidities prolong course of illness. Potential biomarkers have been explored, including hippocampal volumes, neuronal activity of the anterior cingulate cortex, and levels of brain-derived neurotrophic factor (BDNF) and central and peripheral inflammatory markers (e.g., translocator protein (TSPO), interleukin-6 (IL-6), C-reactive protein (CRP), tumor necrosis factor alpha (TNFα)). However, their integration into routine clinical care has not yet been fully elucidated, and more research is needed in this regard. Genetic findings suggest that testing for CYP450 isoenzyme activity may improve treatment outcomes. Strategies such as managing risk factors, improving clinical trial methodology, and designing structured step-by-step treatments are also beneficial. Finally, drawing on existing guidelines, we outline a sequential treatment optimization paradigm for selecting first-, second-, and third-line treatments for acute and chronically ill patients. Well-established treatments such as electroconvulsive therapy (ECT) are clinically relevant for treatment-resistant populations, and novel transcranial stimulation methods such as theta-burst stimulation (TBS) and magnetic seizure therapy (MST) have shown promising results. Novel rapid-acting antidepressants, such as ketamine, may also constitute a paradigm shift in treatment optimization for MDD.

## Depression: a major and relentless burden

Major depressive disorder (MDD) is the most common psychiatric disease and a worldwide leading cause of years lived with disability^1,2^. In addition, the bulk of suicides are linked to a diagnosis of MDD. Despite the high prevalence rate of MDD and ongoing efforts to increase knowledge and skills for healthcare providers, the illness remains both underdiagnosed and undertreated^3^. Many novel strategies with potentially broad impact are not yet ready for ‘prime time’, as they are either in early experimental stages or undergoing regulatory processes for approval. This review sought to: (1) provide a synopsis of key factors associated with outcomes in MDD, and (2) synthesize the existing literature on novel treatment strategies for depression. A literature search was conducted using the search terms ‘depression’, ‘antidepressant’, ‘outcome’, ‘predictor’, ‘(bio)marker’, ‘treatment-resistant depression (TRD)’, and ‘chronic depression’ in addition to combinations of these terms. The search was conducted in PubMed, Scopus, and Google Scholar with no restrictions on time period and concluded in October 2018. Notably, we defined ‘outcomes’ loosely, as either disease course (i.e., treatment resistance, chronic depression) or response/remission to treatment.

## Prognostic variables for treatment outcomes in MDD

### Clinical variables

Clear evidence of an inverse relationship between duration of episode and treatment outcome (either response or remission) underscores the importance of early intervention in MDD^4^ (Table 1). In particular, replicable prospective and retrospective studies indicate that shorter duration of untreated disease—both in terms of first and recurrent episodes—is a prognostic factor indicating better treatment response and better long-term outcomes^5–10^, although not all studies have found such an association^11^. Another important clinical variable is time to antidepressant response. For instance, one meta-analysis found that early improvement was positively linked to antidepressant treatment outcome in 15 of 16 studies^9^. Early response to antidepressant treatment appears to occur independently of treatment modality^12,13^ or outcome parameters^14,15^. Another study found that early improvement in work productivity was a significant positive predictor of higher remission rates after three and seven months of treatment^16^. Similarly, imaging studies found that early response to treatment correlated with default mode network deactivation in the posterior cingulate^17^, as well as thickening of gray matter in the anterior cingulate cortex (ACC)^18^. Interestingly, two recent meta-analyses found that initial improvement was linked to response and outcome but failed to be associated with treatment resistance^19,20^. This suggests that TRD—defined loosely here as non-response to at least two adequate antidepressant trials—and chronic depression (roughly defined here as non-response to any treatment) may have similar response slopes in the earliest treatment stages.

**Table 1:** Candidate markers associated with treatment outcomes

Marker	Outcome	References
Clinical		
Short duration of untreated disease	**↑**	^5– 10^
Early response to treatment	**↑**	^9, 12– 15^
Lower baseline function	**↓**	^21, 22, 24^
Psychiatric comorbidity (anxiety disorders, PTSD, OCD, personality, cumulative)	**↓**	^26, 40– 46^
Physical comorbidity (pain, cardiovascular, neurological, cumulative)	**↓**	^47, 48, 50– 56^
Stressful life events, childhood maltreatment	**↓**	^33– 37^
Treatment resistance	**↓**	^28, 109^
Neuroimaging		
Low baseline hippocampal volume—sMRI	**↓**	^59, 60^
High baseline activity in the anterior cingulate cortex– fMRI, EEG, PET	**↑**	^60, 70, 71^
Microglial activation (TSPO-PET)	**↓**	^80– 82^
rsfMRI in pathophysiologic regions	**↓↑**	^69^
Key proteins of the serotonergic system (MAO-A, SERT, 5-HT_1A_)	**↓↑**	^72– 77^
Blood		
Plasma BDNF increases in response to treatment	**↑**	^93^
IL-6 decreases during treatment	**↑**	^83^
High TNFα levels after treatment	**↓**	^86^
High baseline CRP levels	**↓**	^84, 85^
Candidate genes^a^		
*BDNF*—Val66Met Met allele in Asians	**↑**	^203^
*SLC6A4–5*-HTTLPR, l-Allele	**↑**	^204^

*BDNF* brain-derived neurotrophic factor, *CRP* c-reactive protein, *EEG* electroencephalography, *IL-6* interleukin-6, *OCD* obsessive-compulsive disorder, * PET* positron emission tomography,  *PTSD* post-traumatic stress disorder,  *rsfMRI* resting-state functional MRI, *SLC6A4* solute carrier family 6 member 4, *sMRI* structural MRI, *TNFα* tumor necrosis factor alpha, *TSPO* translocator protein, *5-HT1A* serotonin-1A receptor, *MAO-A* monoamine oxidase A, *SERT* serotonin transporter

^a^Representative examples with meta-analytic evidence

In addition, lower baseline function and quality of life—including longer duration of the current index episode—have been associated with lower remission rates to various types of antidepressant treatments^21,22^. This is in line with results from a previous study that found that baseline function predicted antidepressant response in TRD patients^23^. Worse outcomes in more severely ill patients at baseline were also reported in elderly patients treated in primary-care settings^24^. In contrast, several controlled clinical studies found that elevated baseline severity correlated with improved response and remission rates^25^. Two naturalistic studies with broad inclusion criteria similarly found that remission correlated with higher baseline scores^4,26^. However, this discrepancy might be explained by variations in outcome according to parameter. It was noted earlier that studies that defined remission as percent change of baseline values might be biased in favor of higher baseline scores, while absolute endpoints (e.g., remission defined below a cutoff score) favor less sick patients^4^.

### Psychosocial variables

The influence of sociodemographic factors such as age, age of onset, gender, and number of previous episodes on treatment outcome has been investigated with mixed results^4,27,28^. One study found that females had higher remission rates^21^, but this was not confirmed by another prospective study^27^. Others have found that stress related to high occupational levels might impair outcomes^29^. The European “Group for the Study of Resistant Depression” (GSRD) multi-site study found that age at first treatment (i.e., early-onset and early treatment), age, timespan between first and last episode (i.e., duration of illness), suicidality, and education level were all important variables for outcome^30^. Notably, authors of long-lasting longitudinal studies have suggested that recall bias may influence the age of onset variable^31,32^; given the cognitive deficits associated with acute episodes of MDD, retrospective studies must hence address the factor of memory bias in data collection.

### Environmental stress and stressful life events (SLEs)

High stress levels significantly influence outcomes in MDD patients who are prone to vulnerable states, such as those with high levels of neuroticism^33,34^. A meta-analysis found that history of childhood maltreatment was associated with elevated risk of developing recurrent and persistent depressive episodes, as well as with lack of response or remission during treatment^35^. Another meta-analysis confirmed the detrimental impact of childhood maltreatment (emotional physical or sexual maltreatment or neglect) as a predisposing risk factor for severe, early-onset, and treatment-resistant depression^36,37^. Studies also found gender-specific effects; in particular, at lower stress levels females were at higher risk of MDD than males^34^. Moreover, twin studies have suggested a differential reactivity of gender in response to type of SLE^38^. For instance, a treatment study using escitalopram and nortriptyline investigated the association between number of SLEs (e.g., job loss, psychological trauma, loss of a loved one) and antidepressant treatment. Subjects with more SLEs exhibited greater cognitive symptoms at baseline but not significantly more mood or neurovegetative symptoms. These patients also had greater cognitive symptom reduction in response to escitalopram but not nortriptyline^39^. This suggests that SLEs may have a cognitive domain-specific impact in MDD, but more data are needed to elucidate this issue.

### Psychiatric and physical comorbidities

Psychiatric comorbidity has been shown to influence outcome in both treated and untreated patients^40,41^. Studies have found that elevated baseline anxiety symptoms or comorbid anxiety disorder are associated with worse antidepressant response to first-line selective serotonin reuptake inhibitors (SSRIs) or second-line treatment strategies^42,43^. Worse outcomes have also been reported for MDD patients with comorbid drug or alcohol use disorders, post-traumatic stress disorder (PTSD), and “double depression” (depression and dysthymia)^26,41^. Data from the Sequential Treatment Alternatives to Relieve Depression (STAR*D) study, which included patients who were seeking medical care in routine medical or psychiatric outpatient treatment, indicate that roughly one-third (34.8%) of all MDD patients are free of any comorbidity; the most frequent comorbid Axis-I disorders are social phobia (31.3%), generalized anxiety disorder (23.6%), PTSD (20.6%), and obsessive-compulsive disorder (14.3%)^21^. A large recent study found that clinically diagnosed personality disorder was associated with negative outcomes (with regard to remission and persistent depressive symptoms) six months after diagnosis in MDD subjects enrolled in primary care^44^. Moreover, meta-analytic studies indicate that comorbid personality disorder increases the likelihood of poorer outcomes^45,46^; it should be noted, though, that negative studies have also been reported^40^.

MDD and several physical diseases—including cardiovascular disease and diabetes—appear to have bidirectional effects on disease trajectory^47,48^, yet pathophysiologic links are most likely complex and have to be elucidated. In addition, depression appears to be linked to hormonal diseases, including hypothyroidism^49^. A number of physical disabilities and medical comorbidities have been shown to significantly impact outcome measures in MDD^50^, particularly in elderly subjects^51^. This connection appears to be relevant at any stage of the disease, as number of physical comorbidities did not separate TRD from non-TRD patients^52^. Links between MDD and pain have also been noted; subjects with elevated levels of baseline pain due to chronic conditions had longer depressive episodes, delayed remission^53^ and, most importantly, elevated suicide risk^54,55^. Interestingly, a prospective, 12-month study of older patients found that elderly patients with atrial fibrillation exhibited better remission rates^56^. Patients with chronic pulmonary diseases had worse outcomes in uncontrolled treatment settings than those without these diseases. This difference was absent in the intervention group, in which depression care managers helped physicians with guideline-concordant recommendations and helped patients adhere to treatment^56^. Further longitudinal studies on shared pathophysiology with physical diseases are needed to confirm such associations.

### Neuroimaging markers of treatment outcomes

Structural markers of antidepressant treatment outcomes suggest that hippocampal volumes are related to response and remission^57,58^. One study found that low baseline hippocampal volumes were related to impaired treatment outcomes after 3 years^59^; a meta-analysis confirmed that low baseline hippocampal volumes are associated with negative outcomes^60^. However, negative studies have also been reported^61,62^. The volume of other brain regions, including the anterior cingulate or orbitofrontal cortices, have also been shown to be decreased in MDD subjects^63^, but more longitudinal neuroimaging trials with antidepressants are needed to clarify this association. Interestingly, several studies, including one meta-analysis^64^, found significant hippocampal volume increases after ECT^65–67^, although the relationship to antidepressant response has yet to be confirmed^64,68^.

The largest functional magnetic resonance imaging (fMRI) study of MDD patients conducted to date reported neurophysiological subtypes based on connectivity patterns within limbic and frontostriatal brain areas^69^. In subset analyses, connectivity patterns plus subtype classifications predicted response to repetitive transcranial magnetic stimulation (rTMS) treatment with higher accuracy (89.6%) than clinical characteristics alone. Other task-based and resting-state fMRI studies found that ACC activity (including pregenual activity) predicted treatment response^70^, a finding corroborated by an expanded electroencephalography study^71^ as well as a meta-analysis^60^. While these interesting results suggest that fMRI measures could ultimately help classify biological subtypes of depression, these methods are far from ready for clinical application and results will have to be reproduced. However, given its easy implementation and the short time needed to acquire measurements, fMRI appears to be a promising tool for identifying imaging biomarkers.

**Fig. 1:**
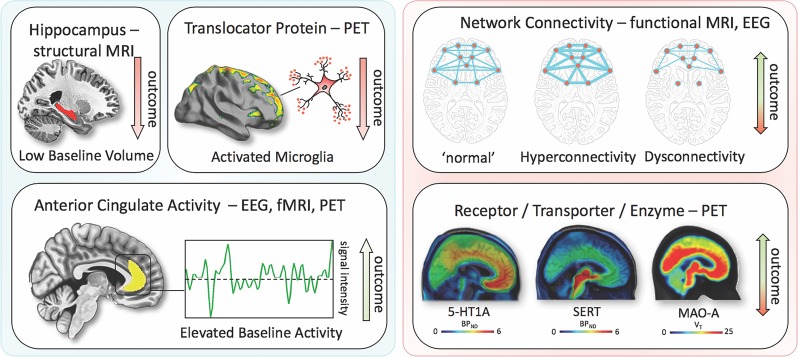
Summary of imaging findings and their relationship with outcome. Imaging findings exhibiting unidirectional (left) relationships with outcome in MDD vs. bidirectional (right). fMRI, functional magnetic resonance imaging; PET, positron emission tomography; EEG electroencephalography; 5-HT1A, serotonin-1A receptor; SERT, serotonin transporter; MAO-A monoamine oxidase-A; BP_ND_, nondisplaceable binding potential; *V*_T_, volume of distribution

Positron emission tomography (PET) studies have identified altered serotonin-1A (5-HT_1A_) receptor and 5-HT transporter (SERT) binding potentials, an index of protein concentration, at baseline and in TRD patients^72–75^. Most of these results found reduced baseline SERT levels and elevated baseline 5-HT_1A_ heteroreceptors in MDD patients (depending on PET methodology for 5-HT_1A_); non-remitters had lower 5-HT_1A_ autoreceptor binding in the serotonergic raphe nuclei^75^, as well as lower SERT^76^. Reduced global 5-HT_1A_ receptor binding has also been observed after ECT^77^. High costs, technical and methodological challenges, lack of dedicated PET centers with ^11^C-radiochemistry, small sample sizes, small effect sizes, and unclear cutoff values have heretofore prevented the broader clinical application of these tools in MDD compared to disorders such as Alzheimer’s and Parkinson’s disease. An earlier [^18^F]FDG PET study of unmedicated MDD patients was consistent with the aforementioned fMRI results, demonstrating increased glucose turnover in the orbitofrontal and posterior cingulate cortices and amygdala and decreased turnover in the subgenual ACC and dorsolateral prefrontal cortex^78^. A later study corroborated these results and found that glucose turnover was differentially affected by cognitive behavioral therapy or venlafaxine^79^. Interestingly, several studies detected microglial activation by labeling translocator protein (TSPO) with PET, using TSPO radioligands like ^18^F-FEPPA. Microglial activation is closely linked to brain tissue damage, traumatic brain injury, neuroinflammation, and increased metabolic demands. Increased TSPO binding in MDD patients has been observed in the ACC, insula, and prefrontal cortex^80^. In addition, TSPO binding has also been shown to positively correlate with length of illness and time without antidepressant treatment, and to negatively correlate with SSRI treatment^80^. Elevated TSPO levels in unmedicated, acutely ill MDD patients have now been reported in at least two independent datasets^81,82^. However, TSPO-positive MDD patients may reflect a specific subtype (i.e., associated with neuroinflammation) and may, thus, respond better to treatments that target neuroinflammation. For a graphical summary of these findings see Fig. 1.

### Blood-based markers of disease outcomes

Consistent with neuroinflammatory processes, elevated levels of C-reactive protein (CRP), tumor necrosis factor alpha (TNFα), and interleukin-6 (IL-6) have been reported in a subset of MDD patients. In particular, elevated levels of CRP, a well-established marker of increased proinflammatory state in blood, was shown to be associated with MDD and increased risk for psychological distress in cross-sectional samples of the general population^83^. A longitudinal study found that lower CRP levels were associated with quicker response to SSRIs, an association not observed for SSRI-bupropion combination therapy^84^. Interestingly, elevated CRP levels have been shown to be more pronounced in female versus male MDD patients^85^. Similar findings have been observed for IL-6 and TNFα. One meta-analysis found that all three were significantly elevated at baseline in MDD patients, but their treatment trajectories differed^86^; IL-6 levels decreased with antidepressant treatment, but outcomes were indistinguishable. In the same meta-analysis, persistingly high TNFα levels identified TRD patients^86^. Notably, heterogeneity was high within the pooled studies. Another study noted that levels of acute phase protein complement C3 significantly differentiated between atypical and melancholic MDD subtypes^87^. MDD patients have also been shown to have altered levels of peripheral adipokines and bone inflammatory markers; these deficits were corrected with ketamine treatment^88,89^.

Given the importance of neuroplasticity in the pathophysiology and treatment of depression, interest has grown in studying brain-derived neurotrophic factor (BDNF), a neurotrophin involved in the structural adaptation of neuronal networks and a prerequisite for neuronal reactions to stressors. BDNF blood levels most likely stem from peripheral tissue. While these peripheral levels are linked to central levels, the question of whether BDNF is actively transported through the blood–brain barrier remains controversial^90^. Compelling evidence suggests that BDNF levels are decreased at baseline in MDD patients and elevated in response to pharmacological^90,91^ treatments as well as ECT^92^. A meta-analysis found that increased BDNF levels in response to treatment successfully stratified responders and remitters compared to non-responders^93^.

### Outcome and genetic and epigenetic links

Heritable risk for MDD is between 30 and 40%, with higher rates in women. A large, collaborative genome-wide association study (GWAS) detected 44 significant loci associated with MDD^94^. Specific analyses identified neuronal genes (but not microglia or astrocytes), gene-expression regulating genes (such as *RBFOX1*), genes involved in gene-splicing, as well as genes that are the targets of antidepressant treatment. The authors suggested that alternative splicing could lead to shifts in the proportion of isoforms and altered biological functions of these proteins^94^.

Hypothesis-driven approaches with candidate genes have provided initial insights into the influence of single-nucleotide polymorphisms (SNPs). It is beyond the scope of this manuscript to review the large number of candidate genes; here, we outline only several representative genes (see Table 1 for meta-analytic evidence of treatment outcomes). These include synaptic proteins involved in stress response, antidepressant binding structures, or neuroplasticity (e.g., CRH receptor 1 (*CRHR1*)), the sodium-dependent serotonin transporter (*SLC6A4*), and *BDNF*^95^. The aforementioned multicenter GSRD study also found that combining clinical and genetic variables explained antidepressant response better than SNPs alone in a random forest algorithm^96^. In that study, regulatory proteins such as ZNF804A (associated with response) and CREB1 (associated with remission), as well as a cell adhesion molecule (CHL1, associated with lower risk of TRD), were linked to antidepressant treatment outcomes. Another interesting candidate gene is FK506 binding protein 5 (*FKBP5*), which was found to moderate the influence of standard treatments in an algorithm lasting up to 14 weeks^97^; *FKBP5* is known to influence HPA axis reactivity^98^, treatment response^99^, and epigenetic mechanisms in response to environmental stressors^100^. Another relevant avenue of research is drug-drug interactions and gene isoforms in the cytochrome P450 pathway (CYP450), which could account for insufficient amounts of a given drug reaching the brain or, conversely, result in exceedingly high plasma values, making subjects more vulnerable to treatment side effects^101,102^. Several commercially available kits categorize patients according to their phenotypic status (e.g., CYP2D6, 2C19, CYP3A4). This led to the introduction of phenotype categories—“poor”, “intermediate”, “extensive (normal)”, and “ultrarapid” metabolizers—based on CYP450 isoenzyme status and their relationship to plasma levels at fixed doses^102^. A large naturalistic study of CYP2C19 isoforms found that treatment success with escitalopram was less frequent in “poor” (CYP2C19Null/Null) and “ultrarapid” metabolizers (CYP2C19*1/*17 or CYP2C19*17/*17)^103^.

### Clinical subgroups, TRD, and treatment outcomes

While some studies have suggested that depressive subtypes in MDD—including anxious, mixed, melancholic, atypical, and psychotic depression—respond differently to antidepressant treatment, this literature is mixed. For instance, some studies found that melancholic patients initially present with high levels of severity and may respond less well to SSRI treatment than to venlafaxine or tricyclic antidepressants^104^, but other studies did not support this finding^105^. No association was found between subgroups and clinical outcomes in a parallel design, uncontrolled study investigating sertraline, citalopram, and venlafaxine^106^, which found that near equal percentages of patients who met criteria for a pure-form subtype (39%) also had more than one subtype (36%), making these psychopathological subtypes difficult to classify.

It should be noted that treatment success might have more discriminatory power for identifying subgroups than psychopathological subgroup stratification. Although a wide range of definitions exists specifying the number of failed trials necessary to diagnose TRD^107^, the core definition of TRD centers around a lack of improvement in response to consecutive, adequate antidepressant treatments. Resistance occurs at alarmingly high rates and is thought to affect 50–60% of all treated patients^107^. Unsurprisingly, this group of patients has dramatically worse outcomes than those who respond to antidepressants, and factors that are associated with TRD overlap with many of those presented above^28^. Cross-sectional data from the GSRD^108^ identified a number of risk factors linked to TRD, including comorbidity (particularly anxiety and personality disorders), suicide risk, episode severity, number of hospitalizations, episode recurrence, early-onset, melancholic features, and non-response at first treatment^28^. Most importantly, TRD is life-threatening, and associated with a two- to threefold increased risk of suicide attempts compared to responding patients, and a 15-fold increased risk compared to the general population^109^. Taken together, the evidence indicates that TRD patients need special attention, as outcomes in these individuals are significantly worse.

## Novel and existing strategies to improve treatment outcomes

### Early identification, prevention, and early treatment

Numerous programs for suicide prevention exist^110^, and recognizing acute depressive symptoms is just one of many important facets of such work. Screening tools for early identification of depressed patients can be helpful^111^, and such instruments can start with as few as two items—for instance, the Patient Health Questionnaire-2^112^ or Ask Suicide-Screening Questions (asQ’em)^113^—and proceed to more detailed instruments if initial screens are positive. Positive screening should be followed by a diagnostic interview to determine whether patients meet criteria for MDD^111^. In the general population, two large independent studies that used only clinical variables were nevertheless able to accurately predict depression within 1–3 years^114^. In addition, long-term monitoring of vulnerable subjects with known SLEs may further improve the ability to identify at-risk individuals early in their course of illness. As noted above, duration of untreated disease is a negative predictor of treatment outcomes. Because the advantages of early intervention in MDD have been demonstrated^115^, efforts to achieve early treatment might also help slow disease progression in individuals with TRD; however, this hypothesis has not been sufficiently tested.

### Modeling environmental impact on predisposition

As noted above, severe SLEs constitute an important risk factor. Elegantly designed studies have demonstrated that genetic predisposition, in concert with SLEs, might account for increased vulnerability to MDD^100^. In this manner, the presence of ‘weak alleles’ in candidate genes such as *BDNF, SERT*, and others would be increasingly detrimental in the presence of SLEs^116,117^. However, studies have been quite inconsistent and yielded small effect sizes, including a negative result in 252 patients enrolled in the GSRD study^118^. It should be noted that counter-regulatory mechanisms or resilience factors, such as social support, may exist that counter SLEs. Nevertheless, preliminary research suggests that the impact of SLEs on MDD may depend on measurable factors such as gender and the timing of exposure^119^. Both genes and the environment are complex systems with frequent opportunity for interaction and elaborate compensatory mechanisms. While the complexity of genetic susceptibility in MDD can be tackled through enormous collaborative projects^94^, the interactions between genetic susceptibility and environmental factors have yet to be determined. Properly powered gene×environment interaction projects may exceed current research capabilities, and large longitudinal studies will certainly be needed^120^.

### Developing markers for subgroup identification and disease course

**Fig. 2:**
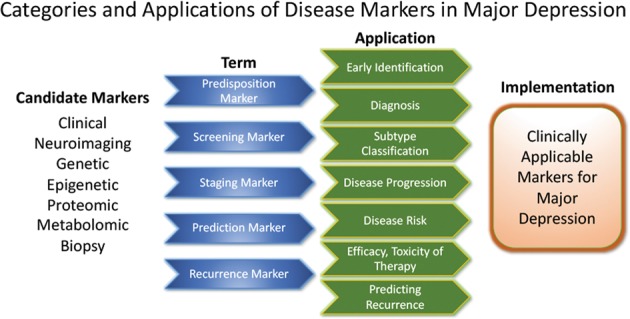
Applicability of candidate markers in MDD. Candidate disease markers can be applied in clinically meaningful ways. While only candidate markers are presently available, sorting these according to their potential applications may facilitate the development of clinically applicable disease markers. The outline follows the classification of markers as suggested by others^200^ (modified and reprinted with permission from Springer)

Pioneering research on biological differences—for instance, between patients with atypical versus melancholic depression—suggests differential HPA axis or autonomous nervous system reactivity^121,122^, though the subtype results have been only moderately consistent across time and are prone to low group specificity^123–125^. However, at least one study demonstrated the more reliable stability of extreme types over a 2-year period^87^. Interestingly, one study found that individuals with atypical depression had significantly higher body-mass index, waist circumference, levels of inflammatory markers, and triglyceride levels, and lower levels of high-density lipid cholesterol than those with melancholic depression or controls^126^. Using fMRI and biological variables, another study found that MDD subjects could be divided into low/high appetite groups with distinctive correlations between neuronal activity and endocrine, metabolic, and immune states^127^. Other research groups have tried to overcome conventional psychopathological subgroups and model biotypes using resting-state fMRI^69^. Molecular and functional neuroimaging, as well as epigenetic studies, are promising approaches for separating subgroups and may be better suited to identifying screening markers (see Fig. 2) that are exclusively valid in certain subgroups with higher predictive power.

These approaches highlight the feasibility of linking and stratifying psychopathological categories with biological variables, a goal further supported by the Research Domain Criteria (RDoc), which seek to link dimensions of observable behavior with neurobiological systems^128^. In the search for biomarkers, subgroup- or domain-specific classifications using unidimensional variables might improve subgroup stratification^129^. Moreover, applying markers to other categories could boost the utility of existing markers that have failed in any given category (see Fig. 2 for established markers). As a field, the focus is largely on staging and prediction markers, but ‘predisposition’ or ‘recurrence’ markers may equally be worth investigating. Presently, however, the relative lack of biologically defined MDD subgroups and their stratification are key obstacles to finding and establishing treament outcome predictors appropriate for broader clinical applications.

The most important outcome of successful subgroup stratification and staging markers would be that patients and their relatives would receive valuable information at treatment onset about how their disease is likely to improve or worsen. Toward this end, the development of staging methods provides promising solutions. Currently, at least five different methods exist^130^ that, to date, have not been evaluated thoroughly enough for clinical implementation. Continuous variables—as obtained by the Maudsley Method and Massachusetts General Hospital Staging Model—appear to provide greater staging advantages than categorical variables. It should be noted here that data indicate that research in severely ill, suicidal, and TRD subjects is safe to conduct in controlled inpatient settings^131^. Presently, patients in various stages of disease and/or treatment history are lumped together and compared in statistical analyses. We propose that staging should be more thoroughly integrated into clinical trial design.

### Algorithm- and guideline-based treatments

**Fig. 3:**
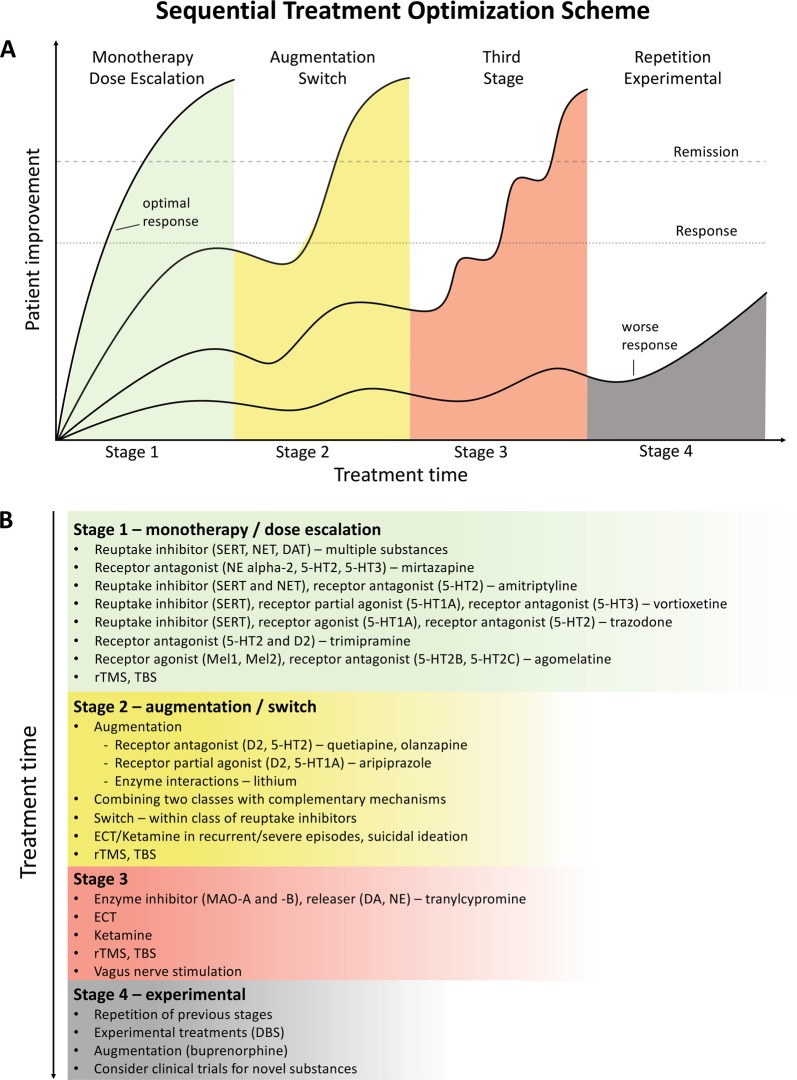
Sequential treatment optimization scheme for major depression. A sequential treatment optimization scheme was generated based on antidepressant treatment guidelines (see Table 2). Treatment optimization is possible for patients being treated for the first time but also for patients with insufficient response to first- or second-stage therapies. **a** Treatment response curves for four common types of patients highlight the importance of sequentially introducing the next step upon non-response to previous steps. **b** Currently available treatments are listed in neuroscience-based nomenclature^201^ with treatment lines corresponding to improvement curves in **a**. Although current classifications vary, patients classified as having treatment-resistant depression (TRD) are eligible for second- or third-stage therapies. 5-HT1A and similar: serotonin receptor subtypes; DBS: deep brain stimulation; DAT: dopamine transporter; D2: dopamine receptor D2; ECT: electroconvulsive therapy; MAO: monoamine oxidase; NET: noradrenaline transporter; SERT: serotonin transporter; TBS: theta-burst stimulation; rTMS: repetitive transcranial magnetic stimulation; DA: dopamine; NE: norepinephrine.

**Fig. 4:**
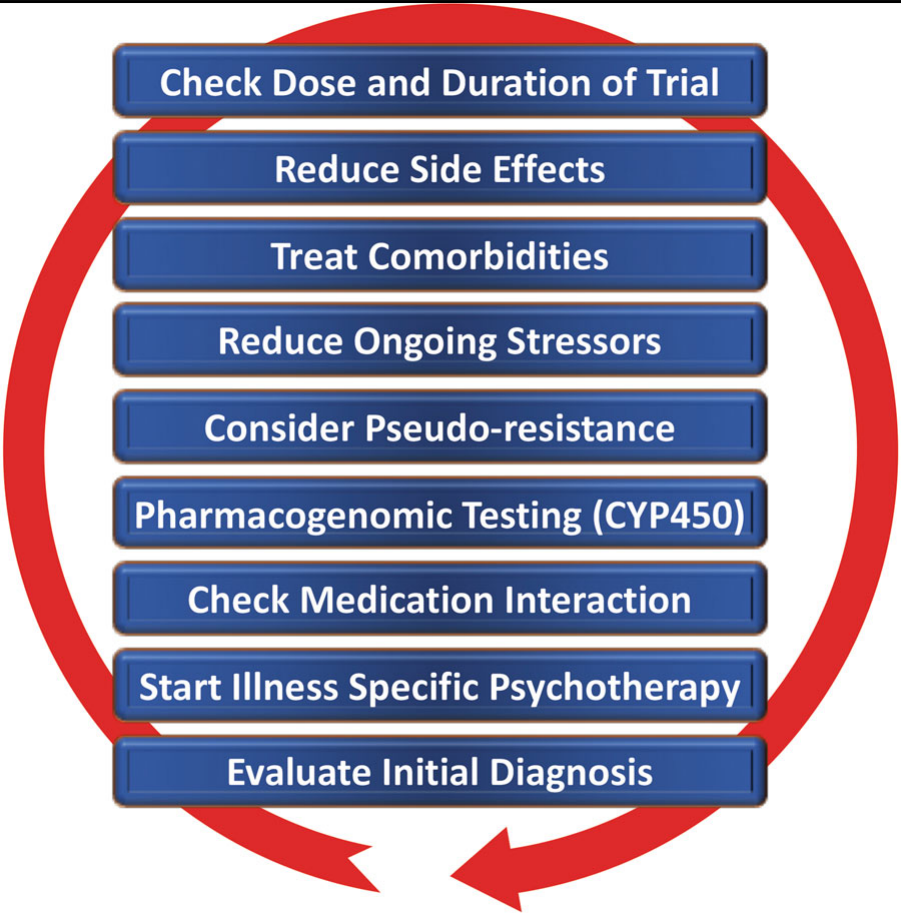
Easily overlooked but efficiently modified factors potentially confounding response to antidepressant treatment (pseudoresistance). Points—in random order—follow earlier suggestions by Dold and Kasper (2017)^202^

Despite the availability and distribution of a variety of expert-based guidelines, only a fraction of patients are actually treated according to guidelines^132^ (see Table 2 for current guidelines (≤10 years)). New guidelines – particularly for TRD – and more rigorous implementation of guideline-based care are needed. Improvements in currently available treatments have been conducted using treatment algorithms and following sequential treatment strategies, with standardized instructions for therapeutic decision-making. In the past two decades, large, collaborative studies using treatment-based algorithms have introduced standardized, sequential treatments; these include the Texas Medication Algorithm Project^133^, the STAR*D trial^21^, and the German algorithm project^134^. Indeed, evidence suggests that algorithm-based treatments improve treatment outcomes^135^ and are cost effective^136^. Here, we considered current clinical treatment guidelines to create a sequential treatment optimization scheme of recommended treatments. While there is no fixed time-frame, first- and second-line treatments are recommended sequentially during the first episode and within 3 months (see Fig. 3, which also illustrates the need for more third- and fourth-stage treatment options). Figure 4, illustrates potential reasons for “pseudoresistance”^42^ that should be ruled out during this time-frame.

**Table 2:** Currently available guidelines and consensus papers

Name/Organization	URL^a^/reference	Country, Year
World Federation of Societies of Biological Psychiatry (WFSBP) consensus papers and treatment guidelines	www.wfsbp.org	Worldwide, 2015, 2013, 2007
American Psychiatric Association Practice Guidelines (APA)	www.psychiatryonline.org/guidelines	USA, 2010
British Association for Psychopharmacology	www.bap.org.uk/guidelines	UK, 2015
Canadian Network for Mood and Anxiety Treatments (CANMAT)	www.canmat.org	Canada, 2016
Institute for Clinical Systems Improvement (ICSI) Healthcare Guideline for Major Depression in Adults in Primary Care	www.icsi.org	USA, 2016
S3 Guidelines	www.leitlinien.de/nvl/depression	Germany, 2017
Therapy resistant depression guideline	www.oegpb.at	Austria, 2017

^a^As of October 2018

**Table 3:** Key points of strategies to improve outcomes in MDD

• Enormous improved outcomes are needed in MDD
• Candidate clinical, neuroimaging, blood, and genetic markers exist but need to be improved to be applicable for routine clinical care
• Early identification and treatment facilitate better outcomes
• The advantages of existing treatments may be harnessed by standardized sequential use
• Novel antidepressants—some with rapid-acting mechanisms—have high potential for approval
• Brain stimulation techniques such as TMS, TBS, ECT, and DBS are evolving and are an important, often underused treatment option
• Treatment strategies for chronic patients exist, but more research needs to focus on “third-line-and-beyond” therapeutics

### Reducing placebo response in clinical trials while harnessing placebo effects in clinical treatment

The issue of placebo response in antidepressant trials has become increasingly important^137,138^. Indeed, the contribution of placebo effects to early response needs to be systematically studied in order to disentangle biological therapy-induced effects from psychologically induced effects. Strikingly, in the brain, anatomically similar regions that mediate placebo response are affected by MDD (for a comprehensive review, see ref. ^139^). Several mechanisms contribute to placebo response, including patients’ expectations of benefits, behavioral conditions, and the quality of patient-physician interactions^139^. Strategies for reducing placebo response could lead to better discrimination between effective treatments in clinical trials; such strategies include extending trial duration, excluding placebo responders by including a placebo run-in, or using randomized run-in and withdrawal periods^138,139^. Others have suggested using more thorough criteria to select study participants^140^. On the other hand, when antidepressant agents are used clinically, placebo effects must be taken advantage of by harnessing patients’ expectations and learning mechanisms to improve treatment outcomes^141^.

### Novel antidepressant treatments

The recent discovery that glutamatergic-based drugs are uniquely capable of rapidly and robustly treating mood disorders has ushered in a new era in the quest to develop novel and effective antidepressants^142–144^. In this regard, the prototypic glutamatergic modulator ketamine has catalyzed research into new mechanistic approaches and offered hope for the development of novel, fast-acting antidepressants. While ketamine’s underlying mechanism of action remains the subject of active investigation, several theories have been propsed^144^. These include N-methyl-d-aspartate receptor (NMDAR)-dependent mechanisms, such as the inhibition of NMDARs on gamma aminobutyric acid (GABA)-ergic interneurons, the inhibition of spontaneous NMDAR-mediated transmission, the inhibition of extrasynaptic NMDARs, the inhibition of lateral habenula neurons, and GABA_B_ receptor expression/function^144^. Substantial evidence also supports additional NMDAR-independent mechanisms, including the stabilization of glutamate release/excitatory transmission, active metabolites such as hydroxynorketamine, regulation of the dopaminergic system, G-alpha subunit translocation, and activation of cyclic adenosine monophosphate, as well as potential sigma-1 and mu-opioid receptor activation^145^. Among those theories, a leading hypothesis remains that NMDAR antagonism increases BDNF synthesis, a process mediated by decreased phosphorylation of eukaryotic elongation factor-2 and the subsequent activation of the mammalian target of rapamycin pathway by BDNF activation of the TrkB receptor^146,147^. These putative mechanisms of action are not mutually exclusive and may complement each other to induce potentiation of excitatory synapses in affective-regulating brain circuits, resulting in improved depressive symptoms.

#### Ketamine

The initial serendipitous discovery that a single, subanesthetic-dose ketamine infusion has rapid-acting antidepressant effects in MDD^148^, a finding subsequently confirmed by numerous randomized trials, has been hailed as one of the most important discoveries in psychiatry in the last decades^149^. The initial proof-of-concept studies demonstrated that a single dose of ketamine (0.5 mg/kg, IV) administered over 40 min led to rapid, robust, and relatively sustained antidepressant effects in TRD—both MDD^150–153^ and bipolar depression^154,155^. In research settings, studies of TRD patients found response rates of >70% within 24 h post-infusion^153^, with about 50–70% of participants exhibiting a variable duration of response^156,157^. Ketamine has also been shown to be superior to any blinding counterpart^158^. Off-label ketamine use has also been associated with significant and rapid (one to four hours) antisuicidal effects^150,159,160^, a finding supported by a large, recent metanalysis showing that ketamine exerted rapid (within hours) and sustained (up to 7 days) improvements in suicidal thoughts compared to placebo^161^.

#### Esketamine hydrochloride

The ketamine enantiomer esketamine received approval by the FDA for TRD and is currently undergoing further Phase III clinical trials. A Phase II, 10-week, clinical trial of flexibly dosed intranasal esketamine (28 mg/56 mg or 84 mg) found that, in TRD patients, this agent demonstrated rapid and clinically relevant improvements in depressive symptoms compared to placebo^162^. Strikingly, 65% of TRD patients met response criteria through Day 57. In another Phase II proof-of-concept, multi-site, 4-week, double-blind study, standard treatment plus intranasal esketamine (84 mg) was compared to standard treatment plus placebo in individuals with MDD at imminent risk of suicide^163^. The authors found a rapid antisuicidal effect, as assessed via the Montgomery-Åsberg Depression Rating Scale Suicide Item score at 4 h.

#### Other rapid acting and novel antidepressants

Based on the success of ketamine, other rapid-acting or novel antidepressant substances within the glutamatergic/GABA neurotransmitter systems are being developed, several of which are in Phase III clinical trials. A prototype novel substance is AV-101 (L-4-cholorkynurenine). This is a potent selective antagonist at the glycine-binding site of the NMDAR NR1 subunit and has demonstrated antidepressant-like effects in animal models, while human Phase II studies are currently ongoing^164^. Brexanolone is a formulation of the endogenous neurosteroid allopregnanolone, which modulates neuronal activation of GABA_A_ receptors and has met positive endpoints in Phase III, leading to FDA approval for postpartum depression. A comparable substance is under development for MDD^165^. In addition, serotonergic agonists have been studied as our understanding of their mechanism of action (e.g., their effects on glutamate release or plasticity) has increased^166^. Encouraging results have been seen for the serotonin 2A receptor agonist psilocybin^167^, but these findings need to be replicated in larger systematic clinical trials. Initial positive trials of add-on agents—such as buprenorphine^168,169^, rapastinel^170^, or scopolamine^145^—have also been conducted. However, it is beyond the scope of this manuscript to review all of these findings, and we refer the interested reader to recent comprehensive reviews of this subject^144,145,165,171^.

### Transcranial stimulation paradigms

In contrast to pharmaceutical treatments that exert their efficacy at the molecular level, electrical stimulation techniques target entire neuronal circuits. TMS of the (left) dorsolateral prefrontal cortex has been FDA-approved since 2008 to treat depression in patients who failed to respond to one standard antidepressant treatment. Apart from transient local skin and muscle irritation at the stimulation site and headaches, it is a very safe technique with few side effects. Studies have repeatedly demonstrated the superiority of rTMS over sham procedures, though effect sizes have been moderate^172–174^. Initial studies suggest that rTMS is also effective in TRD but the data are too few to draw definitive conclusions^175,176^. Improvements in rTMS techniques known as theta-burst stimulation (TBS) provide significantly shortened treatment times (3 min for TBS versus 37 min for rTMS) and hence allow more patients to be treated per day. A large non-inferiority trial of 414 moderately resistant MDD patients found that TBS was at least as effective as rTMS in reducing depressive symptoms^177^.

### Electroconvulsive therapy (ECT)

Regarded as the ‘gold standard’, ECT has been successfully used for many years to treat severe TRD and exhibits both relatively rapid and sustained onset of efficacy; approximately 50% of all patients reach response criteria at the third treatment, typically within 1 week. It is also one of the most effective antidepressant therapies^178^, yielding response rates of ~80%, remission rates of ~75%^179^, and antisuicidal effects^180^. Remission is achieved by about 30% of patients within six ECT sessions^179^. ECT also reduces the risk of readmission^181^ and is likewise safe to use in depressed elderly subjects^182^. The side effects of ECT include intermediate disorientation, impaired learning, and retrograde amnesia, all of which usually resolve^183^. The optimal anatomic location of the stimulus electrodes is a topic of current debate^184,185^. Recent evidence suggests that all three methods for electrode placement (bifrontal, bitemporal, and unilateral) show clinically significant effects^186^. While no difference in cognitive side effects was observed, bitemporal placement should be considered the first-line choice for urgent clinical situations. Despite its clinical efficacy, ECT remains underutilized. Its use is declining^187^ because it needs to be administered in hospital settings under anesthesia, and partly because of misleading portrayals of the procedure itself. Adjusting the dose of electrical stimuli (e.g., through refined electrode placement or individually adjusted pulse amplitudes) may improve ECT’s side effect profile.

### Magnetic seizure therapy (MST)

MST uses high doses of rTMS to induce seizures^188^. The electromagnetically induced electrical field generated by MST is unifocal and variable, as there are individual differences in the degree to which the skull provides electrical resistance^189^. As an advantage over ECT, MST is associated with a more superficial stimulation, which exerts less impact on the medial-temporal lobe where cognitive side effects are thought to be elicited. To date, few research sites across the world have used MST, with a concomitant dearth of open-label trials. Nevertheless, the preliminary treatment data suggest that results obtained with MST are similar to those obtained with ECT but with a more favorable side effect profile^190,191^.

### Vagus nerve stimulation (VNS)

VNS is a surgically implanted pacemaker-like device attached to a stimulating wire threaded along the left vagus nerve. Since 2005, the FDA has approved VNS use for the adjunctive long-term treatment of long-lasting recurrent depression in patients 18 years and older who are experiencing a major depressive episode and have failed to respond to four or more previous adequate standard antidepressant treatment trials. In such cases, it has been shown to have superior long-term effects over conventional psychopharmacological treatment^192^. A recent, large, observational, adjunctive, open-label, naturalistic study followed TRD patients over 5 years^193^. In this group, adjunctive VNS led to significantly better clinical outcomes and higher remission rates than treatment as usual (67.6% vs. 40.9%, respectively).

### Deep-brain stimulation (DBS)

DBS involves the neurosurgical implantation of electrodes and has become clinically routine in the treatment of Parkinson’s disease and Dystonia. The technique is safe, removable, and does not cause lasting neuronal lesions. In TRD, anatomical targets include the subgenual cingulate, nucleus accumbens, habenula, and medial forebrain bundle. Clinical trials typically only enroll severely ill TRD patients whose current episode has lasted >12 months, whose age of onset is <45 years, and who have failed to respond to at least four adequate prior treatment trials of standard antidepressants, ECT, and/or psychotherapy. Initial open-label or single-blind trials found that DBS had both rapid and sustained antidepressant effects^194–196^. In contrast, one large and one smaller sham-controlled clinical study both failed to achieve their primary endpoints of symptom reduction^197,198^. To date, the number of MDD patients treated with DBS has been very small compared to other treatment options, including ECT and TMS. Nevertheless, brain-electrode interfaces are evolving quickly and it is possible that next generation brain-responsive stimulation devices will be able to adjust stimulation on-demand only when abnormal biological marker impulses (e.g., pulse amplitude) are detected^199^.

## Conclusions

Although enormous progress has been made in measuring, predicting, and improving outcomes, depression remains a relentless disease that places a heavy burden on both individuals and society. The research reviewed above indicates that early recognition and early adequate treatment at illness onset are preferable to watch-and-wait strategies. The studies reviewed above also underscore the manner in which SLEs, as well as physical and psychiatric comorbidities, contribute to impaired outcomes. Together, these factors contribute toward treatment resistance, which has gained a substantial amount of importance as a patient-stratifying variable.

This paper also reviewed biological markers, where research has grown exponentially to encompass enormous projects with potentially tens of thousands of subjects enrolled in real world studies. In parallel, studies exploring the underlying genetics of depression have evolved from early candidate gene studies of neurotransmitters, stress, or gene-regulatory systems to large GWAS that help reveal potential new pathways and treatment targets. Moreover, the burgeoning field of proteomics has found promising target molecules. Nevertheless, despite the wealth of recent work in this area, no single biomarker has yet been used in clinical applications. A substantial need exists for replication and, because many biomarker studies are currently open-label, for controlled studies. In combination with neuroimaging techniques such as fMRI, genes or blood-based markers have a high potential of future implementation in stratification of MDD or serve as prognostic marker on treatment outcome.

Above, we also outlined efforts to optimize outcomes. We argue that disease-inherent heterogeneity, in concert with inaccurate group stratification tools, might have contributed to the lack of clinically applicable stratification and response prediction markers. Successful subgroup identification, and the ability to use this information in clinical settings, is crucial to improving future treatment paradigms. While recent research has increasingly focused on TRD, we wish to reiterate that no standard definition of TRD presently exists. Thus, based on currently available guidelines, we have outlined a sequential treatment optimization scheme that includes options for TRD; such work highlights the substantial need to develop and improve “third-line-and-beyond” therapeutics. In this context, this manuscript also reviews novel treatments and brain stimulation techniques that have demonstrated rapid antidepressant effects in TRD, including ketamine, esketamine, ECT, MST, TMS/TBS, VNS, DBS, and others. When treating TRD patients, physicians should consider illness severity, the chronicity of past and recent depressive episodes, the side effect profile of available treatment options, as well as previous refractoriness to particular treatment approaches. If acuity supersedes chronicity, one could consider fast-acting interventions such as ketamine or ECT/MST.

This review, though comprehensive, was not able to consider several lines of evidence on outcome prediction and treatment improvement. In particular, we focused on clinical outcomes in humans and were, thus, unable to fully explore the highly valuable advances made in translational science. Similarly, it was beyond the scope of this manuscript to review the richness of results from animal research and their relevance to MDD. Moreover, given the amount of literature, we were not able to incorporate many proteomic, genetic, or psychopharmacological findings.

Taken together, this review outlines important clinical, psychosocial, and biological factors associated with response and remission to antidepressant treatment (see Table 3). Recent studies have led to important insights into neurobiological disease markers that could result in improved disease stratification and response prediction in the near future. Key discoveries into novel rapid-acting substances, in concert with improvements in brain stimulation techniques, may also result in significantly improved treatment outcomes in formerly hard-to-treat patients.
